# Biofeedback in Partial Weight Bearing: Usability of Two Different Devices from a Patient’s and Physical Therapist’s Perspective

**DOI:** 10.1371/journal.pone.0165199

**Published:** 2016-10-31

**Authors:** Remko van Lieshout, Martijn F. Pisters, Benedicte Vanwanseele, Rob A. de Bie, Eveline J. Wouters, Mirelle J. Stukstette

**Affiliations:** 1 Center for Physical Therapy Research and Innovation in Primary Care, Julius Health Care Centers, Utrecht, the Netherlands; 2 Physical Therapy Research, Clinical Health Sciences & Department of Rehabilitation, Nursing Science and Sport, Brain Center Rudolf Magnus, University Medical Center Utrecht, Utrecht, the Netherlands; 3 Department of Epidemiology, CAPHRI School for Public Health and Primary Care, Maastricht University, Maastricht, the Netherlands; 4 Department of Health Innovations and Technology, Fontys University of Applied Sciences, Eindhoven, the Netherlands; 5 Human Movement Biomechanics Research Group, Department of Kinesiology, KU Leuven, Leuven, Belgium; Universita degli Studi di Perugia, ITALY

## Abstract

**Background:**

Partial weight bearing is frequently instructed by physical therapists in patients after lower-limb trauma or surgery. The use of biofeedback devices seems promising to improve the patient’s compliance with weight-bearing instructions. SmartStep and OpenGo-Science are biofeedback devices that provide real-time feedback. For a successful implementation, usability of the devices is a critical aspect and should be tested from a user’s perspective.

**Aim:**

To describe the usability from the physical therapists’ and a patients’ perspective of Smartstep and OpenGo-Science to provide feedback on partial weight bearing during supervised rehabilitation of patients after lower-limb trauma or surgery.

**Methods:**

In a convergent mixed-methods design, qualitative and quantitative data were collected. Usability was subdivided into user performance, satisfaction and acceptability. Patients prescribed with partial weight bearing and their physical therapists were asked to use SmartStep and OpenGo-Science during supervised rehabilitation. Usability was qualitatively tested by a think-aloud method and a semi-structured interview and quantitatively tested by the System-Usability-Scale (SUS) and closed questions. For the qualitative data thematic content analyses were used.

**Results:**

Nine pairs of physical therapists and their patients participated. The mean SUS scores for patients and physical therapists were for SmartStep 70 and 53, and for OpenGo-Science 79 and 81, respectively. Scores were interpreted with the Curved Grading Scale. The qualitative data showed that there were mixed views and perceptions from patients and physical therapists on satisfaction and acceptability.

**Conclusion:**

This study gives insight in the usability of two biofeedback devices from the patient’s and physical therapist’s perspective. The overall usability from both perspectives seemed to be acceptable for OpenGo-Science. For SmartStep, overall usability seemed only acceptable from the patient’s perspective.

**Implication:**

The study findings could help clinicians to decide which biofeedback device is appropriate for their given situation and provide information for future development of biofeedback devices.

## Introduction

Restrictions of lower-limb weight bearing are frequently instructed in patients after orthopedic trauma or surgery such as lower-limb fractures or osteotomies [1]. Weight bearing (WB) is often restricted to protect the injury site or surgical construct from too much stress that may lead to failure [2–4]. Conversely, the rationale for gradually advancing WB is that repetitive loads can stimulate bone growth and healing [1–3]. Therefore, it is commonly recommended that a rehabilitation program should include WB restrictions, which are gradually reduced as healing occurs [1,5].

Usually, physical therapists (PTs) train patients to comply with WB instructions, using verbal instructions, tactile feedback or bathroom scales [1,6]. However, these methods do not represent dynamic activities (e.g. walking) and are not accurate in training patients to comply with partial weight-bearing (PWB) instructions [5–9]. Previous research shows that it is difficult for patients to comply with WB instructions [1,5,7,9–13]. Reasons for non-compliance include [1] the difficulty to judge the load placed on the lower-limb, and [2] the use of inadequate training methods to achieve controlled PWB [1,7,11,13]. Technological advances have resulted in the development of several commercially available biofeedback devices that are capable of offering real-time feedback on PWB in dynamic situations [1,5,6,11]. These devices intend to enable PTs to assess, train and monitor WB, and aim to provide patients with feedback during daily activities. Examples of such biofeedback devices are SmartStep and OpenGo Science. Both devices are used in supervised clinical settings by PTs and patients and seem promising to improve training and compliance to WB instructions because of providing real-time feedback. These devices use different technology to measure WB and to provide real-time feedback. Thereby, SmartStep has already been used in several studies on PWB [10,14–16]. The recently developed OpenGo Science does not interfere with natural gait by using insoles without external modules attached to the body. Both devices are completely wireless. These two devices were selected based on the findings of earlier research, which showed that criterion validity for peak force measurements under the foot was acceptable in the lower weight-bearing categories for OpenGo Science and SmartStep [17].

Although these devices seem promising, the use of technology in daily practice is often not as successful as expected due to lack of technology acceptance in patients and healthcare professionals [18]. Several studies have outlined that involvement of the user in the development and evaluation of technology is needed for successful implementation [18–20]. Usability of a product has been considered a key aspect of the interaction between user and product [21]. A mismatch among the users’ needs or expectations and the abilities of the biofeedback device can considerably undermine the user-product interaction. It is difficult to precisely define and measure usability [22]. According to the International Organization for Standardization (ISO, 9241–11) usability of a product refers to the extent to which a product can be used by specified users to achieve specified goals with effectiveness, efficiency and satisfaction in a specified context of use [23]. This standard has been widely adopted in the field of usability testing and is commonly referred to as summative usability [22]. Another widely adopted major concept of usability is called formative usability. Formative usability focuses on the detection of usability problems and the design of interventions to reduce or eliminate the impact [22]. In recent decades there have been several extensions to the usability descriptions such as user-centered design (UCD) [22,24] and user experience (UX) [22,25]. Based on previous conceptual considerations De Bleser and colleagues developed a framework specifically for testing the usability of electronic adherence monitoring devices [26]. This framework shows that usability testing of electronic monitoring devices require testing of three aspects: user performance, satisfaction and acceptability. According to the framework, these usability aspects should be tested from a users’ subjective point of view. In the current study the intended users are PTs and their patients after an orthopedic trauma or surgery. Besides involvement of the intended users, usability of the systems should be tested in the specific context in which the biofeedback devices are used, including the tasks users intend to perform.

Therefore, the aim of this study was to describe the usability from physical the therapist’s and the patient’s perspective of SmartStep and OpenGo Science for feedback on partial weight bearing during supervised rehabilitation of patients after orthopedic lower-limb trauma or surgery.

## Methods

### Design

A convergent mixed methods design was used to evaluate the usability of the Biofeedback devices from patient’s and PT’s perspective. Qualitative and quantitative data were collected and analyzed during a similar timeframe [27].

### Participants

Participants consisted of pairs of a physical therapist (PT) and their patient. Participants were recruited by purposive sampling at two different types of physical therapy settings in the Netherlands: (1) private primary care practices and (2) secondary or tertiary care facilities including rehabilitation centers, rehabilitation departments of nursing homes and orthopedic departments of hospitals. In total, nine pairs participated in the study, five from a primary care setting en four from a secondary/tertiary setting. The aim was to recruit a group of participants that was heterogeneous with respect to therapy setting, orthopedic condition, age, sex and education level to facilitate a broad spectrum of usability input [26]. In the current study a sample size of at least four participants per different type of end-user was considered as sufficient. The definition of a sample size for a usability analysis is a complex issue [28]. However, a large number of researchers and practitioners suggest that at least four participants per different type of end-users could be a good starting point to discover at least 80% of a product’s usability problems [22,29–33]. The PTs were eligible if they: trained patients in PWB, worked at earlier described settings and were not influenced concerning the devices by a direct colleague who already participated in the study. Patients were eligible if they fulfilled all the following requirements. They had to be: prescribed with PBW (with a maximum load of 50% body weight) by a doctor because of lower-limb (orthopedic) trauma or surgery; referred to physical therapy; able to walk with crutches; aged 18 years and older; wearing shoes, sized between 38 and 45 (measured by the Continental European System). Patients were excluded when one or more of the following applied: diagnosed with cognitive impairments that prevent the understanding of instructions or the performance of the assigned tasks; not able to wear shoes (e.g. because of edema); having other disorders that interfered with normal gait performance; no good understanding of the Dutch language; diagnosed with central and peripheral neurologic disorders; having a severe hearing impairment (not able to hear the feedback sounds). All participants gave their written informed consent.

### Ethics

The study was approved by the Medical Ethics Research Committee of the University Medical Center Utrecht (date of approval 14 April 2015, METC-protocol number 15-080/C). The study was conducted according to the principles of the Declaration of Helsinki (version: 64th WMA General Assembly, Brazil, October 2013) and in accordance with Dutch acts: Agreement on Medical Treatment Act and the Personal Data Protection Act.

### Biofeedback devices

OpenGo Science (Moticon, Munich, Germany) consists of wireless sensor insoles for data measurement, analysis software for a PC, an ANT radio stick for wireless transmission, and a WB application for a smartphone. Each insole has thirteen pressure sensors, which cover 60% of the insole area, a triaxial acceleration sensor and a temperature sensor. The WB application for the smartphone was a prototype and at the time of the study not commercially available. This application is used to preset an upper threshold for WB and to provide real-time audio or haptic feedback. The analysis software for the PC enables to record and analyze WB data. In live mode, the sensor insoles transmit the data directly to the PC.

SmartStep (Andante medical devices Ltd, Beer Sheva Israel) consists of flexible insoles containing two separate air pockets (one for the forefoot and one for the hind foot). Tubes are used to inflate/deflate each pocket and to connect the pockets to microprocessor control unit, that is worn around the ankle. The microprocessor control unit contains two pressure sensors and is also functioning as a feedback unit by producing an audio signal when a preset WB value is reached. A Software application on a PC is used to preset upper and lower WB thresholds and to record and analyze WB data. In the online mode, SmartStep communicates via wireless Bluetooth USB adapter with a computer.

Table 1 shows an overview of the characteristics of SmartStep and OpenGo Science.

**Table 1:** Characteristics of the biofeedback devices.

	Biofeedback devices	
Characteristics	OpenGo Science	Smart Step
Sensor type	capacitive pressure sensor	silicon pressure sensor
Number of sensors	13	2
Coverage area of the insole	60%	100%
Load range per sensor (unit)	0–40 (N/cm^2^)	0–25 (N/cm^2^)
Feedback unit	smartphone	MCU around the ankle
Connectivity Sensor(s)-FB unit	wireless	via tubes
Feedback type	audio or haptic	audio
Sampling frequency	5 to 100 hertz	40 hertz
Data transfer	wireless or cable to PC	wireless to PC

FB = feedback, MCU = microprocessor control unit, PC = personal computer.

### Data collection

Patient characteristics such as age, gender, educational level, type and location of orthopedic surgery were collected. For PTs the following characteristics were collected: age, gender, physical therapy setting, and experience in instructing PWB.

To evaluate usability, the conceptual framework for testing electronic adherence monitoring devices of De Bleser et al. was used [26]. This framework divides usability from the user’s perspective into three categories: user performance, satisfaction and acceptability. User performance refers to safe and effective use of a device [26]. Satisfaction refers to user-reported advantages, disadvantages, problems experienced when using the device and how much users liked or disliked the device [26]. Acceptability relates to whether the device will be used in the real world [26]. User performance was studied using three methods: (1) counting errors of use during the performed tasks; (2) timing user tasks in seconds; (3) asking participants to think aloud while performing tasks. A think-aloud method is commonly used in usability research, especially when users are confronted for the first time with a device [26,34–36]. This method is used to make explicit what users thoughts and experiences are when performing a specific task. The think-aloud process was videotaped. To investigate aspects of satisfaction and acceptability, patients and PTs were assessed by also using the think-aloud method and by a semi-structured interview. An interview guide with closed questions about satisfaction and acceptability was employed to provide structure to the interviews. During the interview participants were asked to explain their answers. Satisfaction questions were based on the D-quest [37]. Acceptability questions were based on the acceptability concept described in De Bleser et al. [26,38] Semi-structured interviews were audio-recorded.

To evaluate overall usability, patients and PTs were assessed with the System Usability Scale (SUS) [39,40]. This questionnaire consists of 10 statements that are scored on a 5-point Likert scale, ranging from “strongly agree” to “strongly disagree” (scoring range 0 to 4). To calculate the SUS score, the scores of all items are summed and multiplied by 2.5 to obtain the overall SUS score. The overall SUS score ranges from 0 to 100, where higher scores represent better usability. Based on SUS data from a large number of studies Sauro and Lewis produced norms for the interpretation of mean SUS scores, the Curved Grading Scale (CGS) [39,41]. The CGS assigns grades as function of SUS scores and ranges from F (absolutely unsatisfactory) to A+ (absolutely satisfactory), the grades are as follow: Grade F (0–51.7); Grade D (51.8–62.6); Grade C- (62.7–64.9); Grade C (65.0–71.0); Grade C+ (71.1–72.5); Grade B- (72.6–74.0); Grade B (74,1–77.1); Grade B+ (77.2–78.8); Grade A- (78.9–80.7); Grade A (80.8–84.0); Grade A+ (84.1–100). Sauro and Lewis reported the overall mean score to be 68 [39,41]. In the current study, we considered SUS scores of at least 62.7 (corresponding with at least a grade C-) as acceptable usability. The SUS is a simple and reliable method, widely used in usability evaluation [22,39,41,42]. The English SUS shows good internal consistency: between studies Cronbach’s alpha ranged from 0.85 to 0.91 [42}. Construct validity was tested in several studies by factor analysis and showed different factor structures of the SUS [22,39–44]. Originally the SUS intended to be a unidimensional (one factor) measurement of perceived “overall usability”[40,42]. Data from later studies indicated a two-factor structure (with item 4 and 10 aligning on the factor “learnability” separate from de remaining items that aligned on a factor “usability”) [41,43,44]. Recently, Borsci and colleagues found in their study the SUS acting as a unidimensional scale when administered to people who had less product experience but was acting as a bidimensional scale when administered to users with more product experience [41]. In the current study the SUS was considered to be unidimensional because participants lacked experience with the biofeedback devices.

### Procedures

Patients prescribed with partial weight bearing and their physical therapists were asked to use Smart Step and OpenGo-Science during a physical therapy session. So pairs of a patient and a PT could experience these devices. Usability from patient’s and PT’s perspective were assessed separately to prevent bias. In the first session the researcher instructed the patient and the patient underwent the measurements. In the second session the PT instructed the patient and the PT underwent the measurements. The two sessions lasted respectively 60 and 90 minutes. The devices were used in two different equally occurring orders (SmartStep—OpenGo-Science and the reversed order). The pairs were allocated randomly to one of the two testing orders by letting the patient draw blindly one slip of paper without replacement.

### Procedure patient’s perspective

First, measurement procedures were explained to the patient and patient characteristics were collected. Before the usability testing began, patients watched an instructional video to explain the think aloud method based on an example. Patients were asked to use the biofeedback devices one after the other during a training session in which PWB with crutches was practiced. Patients were asked to complete a cluster of specific tasks with the devices. Shortly before executing a particular cluster of tasks, patients watched an instructional video with an explanation of device functions regarding the task to perform. The order of the device presentation was randomised to avoid learning effect and was similar to the allocated testing order. The order of the cluster of tasks was not randomised and occurred in the same order as when patients used the device for the first time.

The clusters of tasks were: putting on the device and using the biofeedback device during PWB. Patients were asked to think aloud when carrying out the tasks. Patients were also encouraged to think aloud by using standardized phrases. The session was videotaped. After completion, the session with the other device started, following the same procedures. Subsequently, patients were asked to fill in the SUS questionnaire per biofeedback device after testing both devices, followed by a semi-structured interview that took approximately 20 minutes.

### Procedure PT’s perspective

In general, the procedures regarding the PT’s perspective were the same as for the patient’s perspective. It also started with an explanation about the measurement procedures, collection of participant characteristics and they also watched instructional videos. Only the PT’s tasks were different from the patient’s tasks: the PT had to program the devices. The clusters of tasks for PTs were: putting on the device, preparing and using the device for instructing PWB, and preparing and using the biofeedback device for monitoring PWB. Subsequently, PTs were also asked to fill in the SUS questionnaire for both biofeedback devices and were interviewed afterwards.

### Data analysis

Descriptive statistics were used to describe participant characteristics and to describe usability measured by (1) the SUS-questionnaire, (2) the interview’s closed questions, and (3) the user performance tests.

Although the primary research aim was to describe and not compare the usability, additional tests (paired samples *t*-tests or the nonparametric Wilcoxon Signed Ranks Tests, both 2-tailed and α = .05) were used to compare the SUS Scores and User Performances for both devices within both perspectives. The assumption of normality was tested with the Shapiro-Wilk test. When the assumption of normality was violated the Wilcoxon Signed Ranks Test was used instead of the t-test.

The video- and audiotaped data from the think-aloud session and the interview were transcribed verbatim. Qualitative data were analyzed using thematic content analysis. Meaningful comments regarding usability were identified and grouped into thematic categories. This was done by one researcher (RL) and checked by a second researcher (MS). For quotations for which no agreement was found, a third researcher (MP) was consulted till consensus was reached among researchers. Qualitative data analysis was conducted with NVivo for the Macintosh version 10.1.1 (QSR International, Doncaster, Australia).

Both quantitative and qualitative data were merged together for analysis and comparison.

## Results

### Participant characteristics

Nine patients (five females) with a median age of 48 years, mostly after hip surgery, participated in this study, together with their PTs (five females). The median age of the PTs was 36 years with a median experience in instructing PWB of 11 years. Characteristics of the study population arranged in pairs of a physical therapist and a patient are presented in Table 2.

**Table 2:** Characteristics of the study population arranged in pairs of a physical therapist and a patient.

	Characteristics						
Pairs	Gender	Age	Physical therapy setting	Years of experience^a^	Educational level patient^b^	Type of surgery	Location of surgery
Physical therapist 1	Male	29	Primary care	4	-	-	-
Patient 1	Male	26		-	Low	Elective	Knee
Physical therapist 2	Male	39	Primary care	14	-	-	-
Patient 2	Female	63		-	Middle	Elective	Hip
Physical therapist 3	Female	31	Primary care	8	-	-	-
Patient 3	Female	44		-	Middle	Elective	Hip
Physical therapist 4	Female	27	Primary care	1	-	-	-
Patient 4	Female	48		-	Middle	Elective	Knee
Physical therapist 5	Female	60	Primary care	38	-	-	-
Patient 5^**c**^	Male	24		-	Low	^**-**^	^**-**^
Physical therapist 6	Male	39	RD of nursing	14	—	-	-
Patient 6	Female	70	home	-	Low	Trauma	Hip
Physical therapist 7	Male	36	OD of hospital	11	-	-	-
Patient 7	Male	59		-	Low	Trauma	Hip
Physical therapist 8	Female	52	OD of hospital	30	-	-	
Patient 8	Female	23		-	High	Elective	Hip
Physical therapist 9	Female	29	Rehabilitation	7	-	-	-
Patient 9	Male	35	Center	-	Middle	Trauma	Femur

RD = rehabilitation department, OD = orthopedic department

^**a**^ Experience expressed in years of instructing partial weight bearing in patients with orthopedic conditions.

^**b**^ A person is defined as low educated if their highest education level is primary education or preparatory secondary vocational education, secondary vocational education, level 1, general secondary education, basic level. A person is defined as secondary educated if their highest education level is higher secondary general education, pre-university education, secondary vocational education level 2, 3, 4. A person is defined as highly educated if their highest education is higher vocational education, university bachelor (BA), university master or PhD level.

^**c**^ This patient had a patella fracture and was treated with conservative treatment (didn’t had surgery).

### Overall perceived usability

The results of the SUS for both biofeedback devices are shown in Table 3. Mean SUS scores of at least 62.7 were considered as acceptable usability and SUS scores were graded according to the CGS as described in the method section. For both SmartStep and OpenGo Science the mean SUS score of patients was above 62.7, i.e., six and eight patients respectively considered the usability as acceptable. The mean SUS score of PTs was below 62.7 for SmartStep and above 62.7 for OpenGo Science, respectively three and eight PTs respectively considered SmartStep and OpenGo-Science as acceptable.

**Table 3:** Perceived overall usability for both biofeedback devices.

	SUS Score		
Biofeedback device	Mean (SD)	CGS Grade	Median (IQR)
*Patients (N = 9)*			
SmartStep	70 (15.9)	C	75 (18)
OpenGo Science^**a**^	79 (12.4)	A-	83 (14)
*Physical therapists (N = 9)*			
SmartStep	53 (17.0)	D	55 (28)
OpenGo Science	81 (10.2)	A	83 (19)

SUS = System Usability Scale, maximum score SUS = 100, SD = standard deviation, CGS Grade = Curved Grading Scale Grade, IQR = interquartile range

Grades range from F (absolutely unsatisfactory) to A+ (absolutely satisfactory) and were assigned as function of mean SUS scores.

^**a**^ OpenGo Science SUS data for patients was not normally distributed.

The SUS scores of SmartStep and OpenGo Science were compared with each other. All distributions of the differences between the devices were normally distributed. The paired sample t-test showed no statistically significant difference in SUS scores between Smart Step and OpenGo Science for the patients, the mean difference was 8.6 (SD = 19.6) points on the SUS (t(8) = 1.3, p = .223). The difference in SUS scores between SmartStep and OpenGo Science for PTs was tested with the paired samples *t*-test as well. The mean difference of 27.5 (SD *=* 14.8) points was statistically significant (t(8) = 5.6, p = .001) in favour of OpenGo Science.

### Categories of usability

#### User performance

Table 4 shows the time registration of the cluster of tasks performed with the biofeedback devices, a description of errors of use, and the number of errors that occurred during the tasks. The results showed that there were not many errors made by the patients. However, four out of nine patients could not attach SmartStep’s control unit around the ankle independently due to hip surgery (hip flexion was not allowed past 90 degrees). Concerning OpenGo Science, initially four out of nine patients wrongly placed the battery in the insole. When looking at the PTs we see that, for SmartStep, five out of nine PTs experienced problems in inflating the air pockets of the insole. After a cue all participants managed to complete this task. For OpenGo Science, there were a lot of connectivity errors between the insoles and the PC or smartphone. Eventually, each participant succeeded in connecting the insoles with the PC and/or smartphone (some with cues or help from the researcher). Additionally, user performance (regarding the timed cluster of tasks) of both devices was compared with each other. All distributions of the differences between the devices were normally distributed. The mean time consumed to put on SmartStep and OpenGo Science by patients was compared with the paired samples *t*-test. The mean difference of 18 (SD *=* 29.1) seconds in favour of SmartStep was not statistically significant (t(5) = 1.4, p = .247). The mean difference between the devices regarding putting on the device by PTs was 102 (SD *=* 59.3) seconds in favour of SmartStep, which is statistically significant (paired samples t-test: t(8) = 5.2, p = .001). The paired samples t-test showed that the difference in mean time used by the PTs to prepare SmartStep and OpenGo Science for use was statistically significant and 363 (SD = 51.5) seconds in favour of OpenGo Science (t(8) = 21.2, p < .001). All PTs managed to prepare SmartStep and OpenGo-Science for use during PWB instructions within respectively 12 and 5 minutes.

**Table 4:** User performances for both the biofeedback devices.

Cluster of tasks		User performance			
	User	Time^a^		Description of errors of use	Errors n^b^ [%]
		Mean (SD)	Median (IQR)		
**SmartStep**					
Putting on the device	Patients	99 [21.4]	101 (37)	Not able to put on the insole and the control unit around the ankle independently because of hip surgery (limited hip flexion was allowed)	4 [44]
				Control unit with tubes was wrongly attached around the ankle	3 [33]
Putting on the device	PTs	208 [44.6]	206 (62)	Air pockets of the insole are not inflated because PT forgot to close the ventil	5 [56]
				Not getting the tubes disconnected from the control unit	1 [11]
				Not getting the manual pump disconnected from the control unit	3 (33)
				Control unit with tubes was wrongly attached around the ankle	3 [33]
Instructing PWB	PTs	546 [72.1]	563 (100)	Connection control unit with PC failed because PT forgot to activate the control unit.	3 [33]
				Investigator had to help PT a lot to remember the steps that has to be performed to use SmartStep during PWB instructions.	2 [22]
				Recording PWB could not be started because PT did not know how to save the patient file	1 [11]
				Patient’s foot was not lifted when PT inflated and calibrated the insole	2 [22]
Monitoring PWB	PTs			-	
**OpenGo Science**					
Putting on the device	Patients	123 [20.8]	128 (15)	Wrongly placing the battery in the insole	4 [44]
				Not able to put on the shoes with insoles independently because of hip injury (limited hip flexion was allowed)	1 [11]
Putting on the device^**c**^	PTs	106 (38.3)	100 (39)	Wrongly placing the battery in the insole	1 (11)
Putting on the device	PTs	106 [71–179]		Wrongly placing the battery in the insole	2 (22)
Instructing PWB	PTs	183 (62.3]	178 (107)	During instructing PWB, accidentally the upper threshold on the smartphone was adjusted.	1 [11]
				During instructing PWB, connection between insole and smartphone was lost because the PT switched from audio to haptic feedback.	1 (11)
				During instructing PWB, connection between insole and Smartphone was lost without a clear reason	1 (11)
Monitoring PWB	PTs			Insoles did not connect with PC because PT forgot to select the right insole size.	7 [78]
				Insoles did not connect with PC because PT forgot to put batteries in both insoles	2 [22]
				Insoles did not connect with PC because PT placed the insoles at the wrong side	2 (22)
				When saving a patient file the software jammed	1[11]
				Initially connection failed because insole was still connected with another host (smartphone), and insole cannot be connected with multiple hosts	1[11]
				During instructing PWB, connection between insole and PC was lost without a clear reason	1(11)

SD = standard deviation, IQR = interquartile range, PTS = physical therapist, PWB = partial weight bearing,— = no errors

^**a**^ Time expressed in seconds

^**b**^ number of errors across all participants

^**c**^ data was not normally distributed.

#### Satisfaction

**Fig 1:**
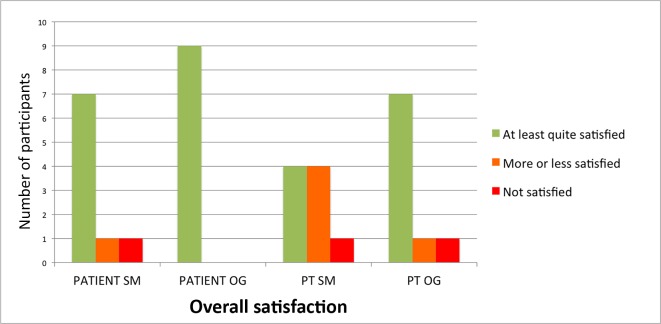
Overall satisfaction with SmartStep and OpenGo Science from the patient’s and physical therapist’s perspective. Patient SM = patient perspective on SmartStep, Patient OG = patient perspective on OpenGo Science, PT SM = physical therapist perspective on SmartStep, PT OG = physical therapist perspective on OpenGo Science.

The results of the satisfaction questions (closed questions) are shown in Table 5. For SmartStep, the quantitative data showed that patients were the least satisfied with ‘ease of use’, ‘overall satisfaction’, and ‘wearable comfort’. Seven out of nine patients were at least ‘quite satisfied’ with ‘overall satisfaction’ and ‘wearable comfort’, and 5 out of nine were at least ‘quite satisfied’ with ‘ease of use’ (see Fig 1). For OpenGo-Science, the data showed that patients were quite satisfied with all examined satisfaction aspects and scored especially high on satisfaction with wearable comfort. All nine patients were at least ‘quite satisfied’ with the “overall satisfaction” (see Fig 1). For SmartStep, quantitative data in Table 5 showed that PTs were the least satisfied with the ‘ease of use’, ‘monitoring patient’s WB’ and ‘overall satisfaction’. Four out of nine PTs were at least ‘quite satisfied’ with ‘monitoring patient’s WB’ and ‘overall satisfaction’, and two out of nine PTs were at least ‘quite satisfied’ with ‘ease of use’ (see Fig 1). For OpenGo Science, the data showed that PTs were the least satisfied with the ‘feedback’ and ‘monitoring patient’s WB’ (for both, five out of nine PTs were at least ‘quite satisfied’). Seven out of nine PTs were at least ‘quite satisfied’ with the ‘overall satisfaction’ (see Fig 1).

**Table 5:** Patient and physical therapist satisfaction with the biofeedback devices measured on a five-point Likert scale.

	Number [%] of participants					
Question	BFD	VS	QS	MLS	NVS	NSA
**Patient Satisfaction**						
1. How satisfied are you with the feedback provided by the device?	SM	1 [11]	7 [78]	1 [11]	0	0
	OG	4 [44]	5 [56]	0	0	0
2. How satisfied are you with the (wearable) comfort of the device?	SM	0	7 [78]	1 [11]	1[11]	0
	OG	6 [67]	2 [22]	1 [11]	0	0
3. How satisfied are you with the ease of use of the device?	SM	1 [17]	4 [44]	3 [33]	1 [11]	0
	OG	3 [33]	5 [56]	1 [11]	0	0
4. How satisfied are you with the degree to which the device helps you to take the right amount of weight bearing (on the affected lower-limb)?	SM	4 [44]	5 [56]	0	0	0
	OG	2 [22]	7 [78]	0	0	0
5. How satisfied are you with the device, overall?	SM	1 [11]	6 [67]	1 [11]	1 [11]	0
	OG	4 [56]	5 [56]	0	0	0
**Physical Therapist Satisfaction**						
1. How satisfied are you with the feedback provided by the device to your patients?	SM	3 [33]	4 [44]	1 [11]	1 [11]	0
	OG	3 [33]	2 [22]	3 [33]	1 [11]	0
2. How satisfied are you with the ease of use of the device?	SM	0	2 [33]	5 [56]	2 [22]	0
	OG	6 [67]	3 [33]	0	0	0
3. How satisfied are you with the degree to which the device helps you with instructing patients in partial weight bearing	SM	2 [22]	6 [67]	0	1 [11]	0
	OG	1[11]	5 [56]	2 [22]	1 [11]	0
4. How satisfied are you with the degree to which the device helps you with monitoring patient’s weight bearing	SM	1 [11]	3 [33]	4 [44]	1 [11]	0
	OG	1 [11]	4 [44]	3 [33]	1 [11]	0
5. How satisfied are you with the adjusting options of the device	SM	2 [22]	3 [33]	4 [44]	0	0
	OG	3 [33]	4 [33]	0	2 [22]	0
6. How satisfied are you with the device, overall?	SM	0	4 [44]	4 [44]	1 [11]	0
	OG	3 [33]	4 [44]	1 [11]	1 [11]	0

*Note*. BFD = Biofeedback device, VS = very satisfied, QS = quite satisfied, MLS = more or less satisfied, NVS = not very satisfied, NSA = not satisfied at all, SM = SmartStep, OG = OpenGo Science.

Satisfaction with the devices extracted from the think-aloud data and the open questions, is illustrated by thematically categorized examples of quotes from patients and PTs shown in S1 Table. The results showed mixed views and perceptions from patients and PTs on satisfaction. A selection of meaningful quotes is presented for each perspective in the text below. After each quote, the participant code and the involved biofeedback device is given (SM = SmartStep and OG = OpenGo Science).

Patients’ perspective

For SmartStep in general, patients were satisfied with the wearable comfort and the effectiveness. This is illustrated by the following quotes:

“It feels comfortable and the materials weigh little, even the control unit with the ankle bracelet”. (patient 4, SM)

“I’m satisfied with SmartStep, it does what it should do.” (patient 2, SM)

Concerning OpenGo Science, in general, patients were satisfied with the wearable comfort, ease of use and intrusiveness. More specific, patients liked the use of normal looking and feeling insoles and the use of a smartphone.

“The insole feels good, you really do not notice that you have it in your shoe”. (patient 1, OG)

“Easy to use system, it is wireless, has no tubes or control unit attached around body parts just insoles and gives feedback via your smartphone, perfect“. (patient 7, OG)

“You hardly notice you are wearing something and that is important. I think it is important that the materials do not bother you too much. You already have enough discomfort due to your operation or rehabilitation.” (Patient 8, OG)

For Smartstep, the views were mixed for satisfaction with the feedback, ease of use and intrusiveness. Some patients experienced SmartStep as an intrusive device because of the control unit around the ankle and the amount of beeps. Furthermore, they disliked the inability to attach the control unit by themselves.

(“I like that the system warns you when you are putting too much or too little weight on the leg. And I’m satisfied with the feedback beeps, the beeps are clear”. (patient 2, SM))

“Attaching SmartStep’s control unit around the ankle is an unhandy activity when you have had hip surgery. I’m not able to attach the control unit by myself”. (patient 3, SM)

“On the one hand it is very nice that you get beeps when you're doing well or aren’t doing well, but on the other hand it is super annoying that the device is beeping constantly, not only for yourself but also for people around you”. (patient 8, SM)

“I think the ankle bracelet around my ankle looks really terrible, it seems a bit like a prison bracelet”. (patient 8, SM)

For OpenGo Science, the views were mixed for satisfaction with feedback and effectiveness. Some patients disliked the impossibility to simultaneously watch the exact amount of loading on the smartphone and walk with crutches and that no WB data was stored on the smartphone.

“Sometimes it is unclear whether you put enough weight on your leg because you are walking with crutches and the smartphone is in your pocket”. (patient 1, OG)

“It is a pity the smartphone did not store data. I can’t look back in the smartphone how much load I placed on my leg. When I walked with crutches and tried to comply with the weight-bearing instructions I could not manage to also look on the smartphone for the kilograms, I only heard the beeps” (patient 7, OG)

Physical therapists’ perspective

Satisfaction from a PT perspective differed for SmartStep and OpenGo Science. For SmartStep, in general, most PTs were at least more or less satisfied with the effectiveness.

“Overall, I’m more or less satisfied. Although it is a cumbersome system it does what it has to do, provide feedback on weight bearing”. (physical therapist 8, SM)

Regarding OpenGo Science, most PTs were satisfied with the wearable comfort, ease of use and intrusiveness. More specific, they liked that the insoles were without external modules attached to the body, and could be connected with a smartphone.

“OpenGo Science is quickly applied, easy to use and no long explanations are needed for patients. There are no wires attached, just put the insoles in the patient’s shoe and ready to go”. (physical therapist 2, OG)

“You cannot see that your patient is wearing a device, great!”. (physical therapist 3, OG)

“The pressure insole looks like a normal insole; it seems comfortable for patients”. (physical therapist 7, OG)

“I am very satisfied with the ease of use. This system is very easy to use. It speaks for itself. It has a good user interface. Furthermore, it is easy to assemble and the insoles can be put easily in the shoes. Thereby it is not uncomfortable for patients, it looks normal, and it uses a smartphone”. (physical therapist 9, OG)

For SmartStep, PTs expressed mixed feelings concerning feedback, wearable comfort, ease of use and intrusiveness. Especially, comments on SmartStep’s feedback varied at lot. Some PTs liked that it was possible to preset a lower and upper threshold, whereas others had some concerns about the auditory feedback. With respect to the ease of use, more than half of the PTs said that there were a lot of steps before they could actually use SmartStep.

“It is quite a device and it is really visible. When used in practice is not necessarily an issue but used outside it is”. (physical therapist 3, SM)

“I'm satisfied with the feedback. I like that not only feedback is provided when the patient is loading too much but also when the patient is loading correctly. It encompasses the patient and the physical therapist”. (physical therapist 5, SM)

“Too much auditory information, beeps, not pleasant for me as a physical therapist and for the patient” (physical therapist 6, SM)

“I’ am not satisfied with the ease of use. I think it is a cumbersome system because of the following things. The control unit around the ankle, the tubes and inflation procedure and you have to add an insole to one of shoes and people have often already swollen feet due to surgery”. (physical therapist 8, SM)

PTs perceptions were also mixed with respect to OpenGo Science’s feedback and effectiveness. Most PTs liked that both audio and haptic feedback was possible. Some PTs disliked that a feedback signal was only provided when the upper threshold was exceeded and most PTs regret that feedback via smartphone was not available during recording WB via the PC.

“The ability to provide audio and haptic feedback as well is nice. It is a calm sound and you change the feedback to vibrations so only the patient is provided with the feedback”. (physical therapist 3, OG)

“When recording patient’s weight bearing the biofeedback from the smartphone is not available, I would have preferred to also use the smartphone’s feedback during the measurements”. (physical therapist 6, OG)

“I had to look a lot on the display of the mobile. I would like to have more auditory information for the patient. The system did give a beep when the patient loaded the leg too much, but didn’t beep when the patient loaded properly or loaded the leg to little. Therefore, I am more or less satisfied with the feedback”. (physical therapist 8, OG)

#### Acceptability

The results of the closed acceptability questions are shown in the Table 6. In this section only the main acceptability results are presented. Regarding acceptability from the patient’s perspective, the results showed that eight out of nine patients answered the question whether they would recommend SmartStep to other people in a similar situation (prescribed with PWB) with at least ‘probably’. For OpenGo-Science all patients answered this question with at least ‘probably’. Looking at willingness to pay a contribution for the biofeedback device, patients responded more unfavorably concerning SmartStep in comparison with OpenGo-Science. In the former case six out of nine patients answered with at least ‘probably’, whereas in the latter case eight out of nine patients responded with at least ‘probably’. Regarding acceptability from PTs’ perspective Table 6 shows the following: Two out of nine PTs would recommend SmartStep at least ‘very probably’ to colleagues and seven out of nine PTs would recommend OpenGo-Science ‘very probably’ to colleagues. Furthermore, five out of nine PTs wanted to purchase SmartStep at least ‘probably’ and eight out of nine PTs wanted to purchase OpenGo-Science at least ‘probably’. Reasons for non-acceptance of SmartStep emerging from the interview were: complexity of the device, intrusiveness of a control unit around the ankle, intrusiveness of the audio feedback and the availability of more usable devices. The reason for non-acceptance of OpenGo Science experienced by one PT were the encountered connectivity problems between the insole and the smartphone.

**Table 6:** Patient and physical therapist acceptability of the biofeedback devices measured on a five-point Likert scale.

	Number [%] of participants					
Question	BFD	D	VP	P	PN	VPN
**Patient Acceptability**						
1. Would you recommend this device to other people in your situation?	SM	2 [22]	3 [33]	3 [33]	1 [17]	0
	OG	0	6 [67]	3 [33]	0	0
2. If you are in a similar situation in the future, do you intend to use this device again in supervised rehabilitation	SM	2 [22]	7 [78]	0	0	0
	OG	0	8 [89]	1 [11]	0	0
3. If you are in a similar situation in the future, do you intend to use this device also in the rehabilitation at home	SM	2 [22]	3 [33]	2 [22]	0	2 [22]
	OG	3 [33]	4 [44]	1 [17]	0	1 [11]
4. Would you be willing to pay a contribution for the use of this device	SM	2 [22]	2 [22]	2 [22]	1 [11]	2 [33]
	OG	4 [44]	1 [11]	3 [33]	1 [11]	0
**Physcial Therapist Acceptability**						
1. Would you recommend your colleagues to use the system	SM	0	2[22]	5[56]	2 [22]	0
	OG	0	7[78]	1 [11]	0	1 [11]
2. Would you like to use this system in the future in the supervized rehabilitation	SM	0	3 [33]	3 [33]	3 [33]	0
	OG	0	6 [67]	2 [22]	0	1 [11]
3. Would you like to use this system in the future in the home rehabilitation	SM	1 [11]	0	2 [22]	4 [44]	2 [22]
	OG	0	4 [44]	2 [22]	2 [22]	1 [11]
4. Do you want to purchase this device in the future for use it your patients?	SM	0	2 [22]	3 [33]	3 [33]	1 [11]
	OG	0	4 [44]	4 [44]	0	1 [11]

*Note*. BFD = Biofeedback device, D = Definitely, VP = Very Probably, P = Probably, PN = Probably Not, VPB = Very Probably Not, SM = SmartStep, OG = OpenGo Science.

## Discussion

This study described the usability from the physical therapist’s and patient’s perspective of the biofeedback devices, SmartStep and OpenGo Science when used for feedback on PWB during supervised rehabilitation after lower-limb trauma or surgery. In general, the overall usability measured on the SUS suggested that both SmartStep and OpenGo Science were at least acceptable from the patients’ perspective. From the PT’s perspective OpenGo Science seemed to be acceptable and SmartStep seemed not to be acceptable.

Looking at user performance, the results showed that there were not many errors made by the patients. However, more than one-third of the patients could not attach SmartStep independently due to hip surgery. Concerning OpenGo Science, more than one-third of the patients wrongly placed the battery in the insole of OpenGo Science. All PTs managed to prepare SmartStep within twelve minutes and OpenGo-Science within five minutes for use during PWB instructions. Five minutes seems more acceptable instead of the twelve minutes since most PTs have treatment sessions of 30 minutes per patient. Furthermore, the majority of the physical therapist had problems with inflating the air pockets of SmartStep while OpenGo-Science in some cases encountered connectivity problems. Eventually, all problems of the physical therapists were solved with cues or help from the researcher. It should be noted that time spent to prepare the devices and found usability problems, are expected to decrease when patients and PTs have more experience in using the devices. Problems that seem to be of a more structural nature were: attaching SmartStep by patients after hip surgery and some of OpenGo Science’s connectivity issues experienced by the physical therapists.

Looking at satisfaction, there are mixed views and perceptions from patients and PTs. In general, the majority of the patients were overall at least quite satisfied with the biofeedback devices. For SmartStep, the quantitative data showed that patients were the least satisfied with ‘ease of use’, ‘wearable comfort and ‘overall satisfaction’. This could be explained by the qualitative data, some patients experienced SmartStep as an intrusive device because of the control unit around the ankle and the amount of beeps. Some patients disliked the texture of the insole, the multiple operating steps and that they were not capable to attach the control unit by themselves due to hip surgery. This is in line with Fu et al. who mentioned that SmartStep is besides its effectiveness, a complex and auditory intrusive device [5]. Concerning OpenGo-Science, the quantitative data showed patients were quite satisfied with all examined satisfaction aspects. Patients scored especially high on satisfaction with ‘wearable comfort’. This is in line with the qualitative data showing that all patients expressed positive feelings regarding the ‘wearable comfort’ of the insoles. PT satisfaction differed more than patient satisfaction. In general, concerning the overall satisfaction (quantitatively measured), PTs were more positive about OpenGo Science. Less than half of the PTs were at least quite satisfied with SmartStep and more than three-quarters of the PTs were at least quite satisfied with OpenGo Science. The qualitative data suggests that especially satisfaction with ‘wearable comfort’, ‘ease of use’ and ‘intrusiveness’ contributed to this good ‘overall satisfaction’ with OpenGo Science. For SmartStep, satisfaction with ‘effectiveness’ seemed to contribute positively to the ‘overall satisfaction’. Moreover, for SmartStep, quantitative data showed, that PTs were the least satisfied with ‘ease of use’, ‘monitoring patient’s WB’ and with ‘overall satisfaction’. This might be explained because the majority of the PTs indicated that there were a lot of steps before they actually could use SmartStep and besides that they disliked the intrusiveness of the device (e.g. amount of beeps heard and control unit around the ankle). Furthermore, as disadvantage the short maximum recording time of approximately 10 minutes was mentioned. Regarding OpenGo Science, the quantitative data showed that most PTs were the least satisfied with the ‘feedback’ and ‘monitoring patient’s WB’. This might be explained by more than half of the PTs who disliked that feedback via smartphone was not available during recording WB via the PC; and more than half of the PTs disliked that patients are only provided with feedback when they were loading the affected leg too much and not when they were loading properly or too little.

Looking at acceptability, in general, patient acceptability for SmartStep and OpenGo Science for use during supervised rehabilitation was good. When looking at PT acceptability, the data about intention to purchase in the future suggested poor acceptability for SmartStep and good acceptability for OpenGo-Science. Thereby, it should be noted that PTs were not informed concerning the real price of SmartStep and OpenGo-Science. This could have influenced the acceptability because high costs might undermine acceptance, as being a critical determinant of technology acceptance [45]. Reasons for non-acceptance of SmartStep by the PTs emerging from the interview were: complexity of the device, intrusiveness of a control unit around the ankle, intrusiveness of the audio feedback and the availability of more usable devices.

The quantitative satisfaction data suggested that patients and PTs were mostly satisfied with the feedback provided by the biofeedback devices. However, during the think-aloud sessions and the interviews patients and PTs expressed different needs and expectations about feedback. For example, some participants preferred to receive only feedback when they exceeded an upper WB threshold, others preferred to receive also feedback when WB was within the target zone. Perhaps, the limited feedback options of the devices don not match with the different users’ needs and expectations. Previous studies indicate that biofeedback devices have to be flexible in the way that they are able to adapt to users’ feedback needs and learning phase [46,47]. Sigrist et al. suggested in their theoretical review that motor learning should start with real-time feedback during the motor task execution [46]. Subsequently, real-time feedback should be switched to a lower frequency or changed to postponed feedback to facilitate automation of the movement [46]. Furthermore, they suggested that self-controlled feedback offers a possibility for adapting feedback to the current phase of the learner. Therefore, it highly involves and motivates the learner and may also promote self-efficacy [46]. Winstein et al. studied the effectiveness of real-time and post-response feedback in learning PWB skills [47]. They suggested that practice with real time feedback is beneficial for immediate performance, but not for learning of PWB skills. For long-lasting learning the skill, post-response feedback is more effective [47]. Moreover, a recent review on using feedback through digital technology to increase performance identified multiple factors moderating feedback efficacy. These factors are receiver traits & states, feedback properties such as technology, content, timing, modality, duration, frequency, presentation, and user experience [48]. Although there is emerging evidence, research on the effect of feedback through digital technology has just started and the optimal choice of feedback properties for effective feedback interventions remains unclear [48].

Strengths of the study were that the intended users (PTs and patients prescribed with PWB) were involved and usability was tested in the specific context in which the biofeedback devices should be used. Hereby, a clear view was provided what users experiences were when using the different devices in the intended context of use. Another strength was that usability was evaluated with the conceptual framework for testing electronic adherence monitoring devices of De Bleser et al. [26]. This was the most specific and applicable framework found for testing usability of electronic monitoring devices and it builds on existing literature and frameworks [26,49,50]. In this framework several qualitative and quantitative evaluation methods have been proposed. Mixing qualitative and quantitative evaluation methods ensures comprehensive data collection and avoids needless a priori assumptions [26,34,36]. However, there are also limitations of using the conceptual framework of De Bleser et al. This framework is not widely adopted in usability testing and although it builds on existing literature and frameworks it remains unclear how valid this conceptual framework itself is. Another limitation could be the short time participants worked with the devices. Although the authors think that evaluating the first experiences is information-rich, identifies most of the usability issues and tests the learnability of the devices (devices should be simple enough for novel users to learn its functions easily), the authors also realizes that this short time may have affected the results. In this study all users were inexperienced and had no user experience with the tested biofeedback devices. More time with the devices may result in a better user performance or effectiveness and a more positive user satisfaction. For instance, AL-Maskari’s study showed that better user performance resulted in a greater user satisfaction [51]. Additionally, it should also be noted that testing the two devices in one session may have influenced the results. Although participants were asked not to compare the devices much of the participants verbally used the other device as reference in the usability testing. This could have affected the usability negatively or positively depending of the superiority or inferiority of the reference device. To prevent this bias as much as possible, explicit instructions were given to participants (prior and during the usability testing) not to compare the devices directly. Furthermore, in the current study inferential statistics were used to compare the SUS scores and user performances regarding both devices. However, it was not the primary research aim to compare the devices and the study was not specifically designed for a comparison (see e.g. the small sample size and the sample size determination). Therefore, one should be cautious in drawing conclusion based on the inferential statistics.

The findings of the current study could help clinicians to decide which biofeedback device is appropriate for their given situation and provide information for future development of biofeedback devices. This study only provides information on the usability during supervised rehabilitation. However, there is a growing need from clinical practice and research for biofeedback devices that provide real time biofeedback and can collect data in daily life for monitoring purposes. Further research should investigate the usability of biofeedback devices for monitoring purposes in patients’ own home and community setting.

## Conclusion

The results of the current study give insight in the usability of two biofeedback devices from the patient’s and physical therapist’s perspective when used in supervised rehabilitation of patients after lower-limb trauma or surgery. The overall usability of SmartStep and OpenGo Science seemed acceptable from the patient’s perspective. From the PT’s perspective OpenGo Science seemed to be acceptable and SmartStep seemed not acceptable. The study findings could help clinicians to decide which biofeedback device is appropriate for their given situation and provide information for future development of biofeedback devices.

## Supporting Information

S1 Table Thematically categorized examples of patients’ and physical therapists’ comments on the usability of biofeedback devices extracted from the think-aloud data and the open questions. *Note*. PT = physical therapist.
(DOCX)
