# Supplementary material for: Biofeedback in Partial Weight Bearing: Usability of Two Different Devices from a Patient’s and Physical Therapist’s Perspective
Source: PLoS One. 2016 Oct 31;11(10):e0165199. doi: 10.1371/journal.pone.0165199 (PMC5087887; doi:10.1371/journal.pone.0165199)
Supplement: S1 Table — Note. PT = physical therapist. (DOCX) [file pone.0165199.s001.docx]

**S1 Table. Thematically categorized examples of patients’ and physical therapists’ comments on the usability of biofeedback devices extracted from the think-aloud data and the open questions.**

| **Thema** | **Comment (type of participant and number)** |
| --- | --- |
| **Feedback** | ***SmartStep*** |
|  | - “You are getting a clear signal when you are loading according to the target load, that is good”. (patient 1) - “I like that the system warns you when you are putting too much or too little weight on the leg. And I’m satisfied with the feedback beeps, the beeps are clear”. (patient 2) - “Though feedback beeps are clear and work properly, I do think that haptic feedback is more convenient than a beep. Especially when you go outside”. (patient 3) - “I’m satisfied with the feedback; it clearly signals when you exceed your weight-bearing limits”. (patient 4) - “I’m satisfied with the feedback. I can hear the feedback beeps clearly. But I can imagine not everyone can hear the beeps. Perhaps it would be useful too if the control unit would provide short vibrations to the leg or ankle”. (patient 5) - “I felt that the numerous feedback beeps were disturbing”. (patient 6) - “On the one hand it is very nice that you get beeps when you're doing well or aren’t doing well, but on the other hand it is super annoying that the device is beeping constantly, not only for yourself but also for people around you”. (patient 8) - “I’m satisfied with the degree to which the device helps me to take the right amount of weight bearing. It clearly shows the amount of weight bearing. Normally, I don’t have a clue “. (patient 9) - “More or less satisfied with the feedback. No beep, single and double beeps give a direction to the patient, respectively increasing, maintaining or decreasing weight bearing, that seems absolutely useful. But the difference between a single and a double beep is maybe difficult to hear for elderly and persons with hearing problems”. (PT 2) - “I’m satisfied with the feedback. It is an advantage that you can preset a lower and an upper weight-bearing threshold. The feedback sounds are clearly heard. But you are hearing a lot of beeps and the sounds are very annoying. You even hear one beep when you are loading properly. I think this can be demotivating to patients”. (PT 3) - “It is convenient that the system gives immediate audio feedback by a beep, you don’t have to look at something else”. (PT 4) - “I’m very satisfied with the feedback. It clearly indicates when patients put too much or too little weight on their feet. You can train the patient in the target weight-bearing zone very well”. (PT 4) - “I'm satisfied with the feedback. I like that not only feedback is provided when the patient is loading too much but also when the patient is loading correctly. It encompasses the patient and the physical therapist”. (PT 5) - “Too much auditory information, beeps, not pleasant for me as a physical therapist and for the patient” (PT 6) - “If someone is loading the leg properly, you hear with every step a beep. I think SmartStep beeps too much. Perhaps usability is better when SmartStep only beeps depending on whether the lower or upper threshold is exceeded.” (PT 7) - “The feedback is very nice, patients are provided with instant feedback. They immediately know if weight bearing is within the target zone, or too much or too little”. (PT 8) - “I am satisfied with the feedback because you can set upper and lower thresholds. And the feedback is real time, patients can immediately react to it”. (PT 9) |
|  | ***OpenGo Science*** |
|  | - “This device beeps too quickly, I deliberately try to put little weight on my leg but it still beeping frequently”. (patient 2) - “The feedback sounds are clear”. (patient 6) - “I’m very satisfied with the feedback because it tells me when I am loading my leg to much and I can hear the beeps clearly”. (patient 4) - “The system gives only a beep or vibration when I am loading to much and that’s good. It would be irritating when you constantly hear beeps”. (patient 8) - “I am satisfied with the feedback, it works fine, supposing that the feedback is accurate”. (patient 9) - “The system gives immediate feedback to the patient when she/he is loading too much. That is quite convenient. I would have liked when it also provided feedback if the patient loaded the leg to little”. (PT 1) - “For patients it is clear that beeps are only heard when the upper weight-bearing threshold is exceeded. With this patient the beeps were a bit demoralizing because with almost every step the patient exceeded the threshold so the beeps were continuously present” (PT 2) - “The ability to provide audio and haptic feedback as well is nice. It is a calm sound and you change the feedback to vibrations so only the patient is provided with the feedback”. (PT 3) - “Perhaps it is a pity when using the smartphone the patient does not know the exact amount of weight bearing or threshold exceedance, Namely, the smartphone is in the patient’s pocket and the sounds do not give an indication”. (PT 3) - “I’ am more or less satisfied with the feedback, the beep is clear but I regret you cannot set a lower weight-bearing limit. Therefore, it is difficult to immediately conclude that your patient has not put enough weight on the foot”. (PT 4) - “I’m not satisfied with the feedback. Occasionally there was a signal loss and no feedback on weight bearing was provided, which is very regrettable. In that case, it doesn’t do what it is supposed to do”. (PT 5) - “I like the audio feedback, especially that it is only present when the patient puts to much load on the leg”. (PT 7). - “I had to look a lot on the display of the mobile. I would like to have more auditory information for the patient. The system did give a beep when the patient loaded the leg too much, but didn’t beep when the patient loaded properly or loaded the leg to little. Therefore, I am more or less satisfied with the feedback”. (PT 8) - “I am more or less satisfied. The feedback is very clear, but I would have preferred the possibility to set upper and lower weight-bearing thresholds”. (PT 9) - “You will hear the same kind of feedback beeps when the patient has slightly or largely exceeded the upper weight-bearing threshold. It would be nice if you could also set a lower threshold and get an indication how much too low or too high your patient is loading the leg because you want to optimize weight bearing. Perhaps something can be done with the audio signal”. (PT 9) |
| **Wearable comfort** | ***SmartStep*** |
|  | - “I don’t even feel that there is an insole inside the shoe”. (patient 2) - “The insole feels fine in the shoe. you can barely feel it”. (patient 3) - “I’m not satisfied with the wearable comfort; the insole doesn’t feel very pleasant. The bumpy insole is irritating my foot”. (patient 8) - “It feels comfortable and the materials weigh little, even the control unit with the ankle bracelet”. (patient 4) - “Insole and control unit around the ankle fits good”. (patient 6) - “I am more or less satisfied with the wearable comfort, basically because of the shape and the texture of the insole. Besides that your foot is placed higher in your shoe by adding the insole, it feels less comfortable.” (patient 9) - “Altogether, it works fine. However, it would be nice if the insole feels a bit more comfortable and natural”. (patient 9) - “I can imagine that I device around the ankle is cumbersome, it is also relatively large especially when the patient has thin legs”. (PT 6) - “The insole is inserted fairly easy, but I have the impression the insole is not fitting properly in the shoe”. (PT 6) - “The insole is not so comfortable for the patients, that is a disadvantage”. (PT 7) |
|  | ***OpenGo Science*** |
|  | - “The insole feels good, you really do not notice that you have it in your shoe”. (patient 1) - “You hardly notice that you have an insole in your shoe”. (patient 3) - “I’m very satisfied with the wearable comfort. It feels good and you are not aware of wearing a system.” (patient 4) - I’m satisfied with the wearable comfort. The only downside was that I couldn’t remove the insoles of from my sport shoes, the shoes become a bit tight by the insoles”. (patient 5) - “Insoles feel comfortable”. (patient 6) - “The insoles look and feel like normal shoe inlays”. (patient 7) - “The pressure insole looks like a normal insole, it seems comfortable for patients”. (PT 7) |
| **Ease of use** | ***SmartStep*** |
|  | - “There are a lot of actions you have to perform before you can use SmartStep”. (patient 1) - “It is a disadvantage that I need some help with putting the device on. Due to hip surgery I’m not able to flex the hip so far and cannot put it on by myself”. (patient 2) - “Attaching SmartStep’s control unit around the ankle is an unhandy activity when you have had hip surgery. I’m not able to attach the control unit by myself”. (patient 3) - “I’m satisfied with the ease of use, it is not complicated to use or wear”. (patient 4) - “I’m more or less satisfied with the ease of use. Perhaps some things could be made easier”. (patient 5) - “It is not easy to put on the control unit because I have a mobility limitation in my hip”. (patient 7) - “I’m not satisfied at all with the ease of use because I can’t put it on my own due to hip surgery and that’s not convenient. I can’t count on somebody who can help me me in all situations”. (patient 8) - “SmartStep is easier to use then I expected based on the way it looks”. (PT 1) - “I think that putting on the device and presetting the device is too time consuming”. (PT1) - “I can preset a lower and upper threshold, and I can look back in the analysis software precisely how someone loaded the leg regarding the thresholds, it's really nice system”. (PT 1) - “Attaching the tubes with the control unit and placing the insole in the shoe is easy to do”. (PT 2) - “First impression of the analysis software is that is looks practical”. (PT 2) - “I like the compliance diagram in the software. This summary can help you to stimulate patients to achieve better compliance rates” (PT 3) - “You have to undertake quite a few actions before you can start using the system, such as pumping up the insole, attaching the control unit, connecting the insole…….. These actions cost considerable time“. (PT 3) - “I’m satisfied with the ease of use. Although there are quite a few steps before you can use it, it's doable. I think, when you know this device better and have worked a couple of times with it, it will be easier and easier”. (PT 4) - “I’m more or less satisfied with the ease of use. I didn’t like the many components that had to be connected differently, it wasn’t easy to put the insole in the shoe, and there were a lot of steps such as inflating the insole. That is not so handy”. (PT 5) - “Too many actions before you can actually use de feedback device, not easy to use”. (PT 6) - “The analysis software is informative, it works fast, gives a clear reflection of the weight bearing and it is positive that weight bearing is expressed in kilograms”. (PT 6) - “Usability of the analysis software is super, you can see how the patient is responding to the audio feedback, and you can watch the saved weight bearing data”. (PT 7) - “With the control unit and those tubes attached you have to watch out that you don’t stumble or damage the device”. (PT 8) - “I’ am not satisfied with the ease of use. I think it is a cumbersome system because of the following things. The control unit around the ankle, the tubes and inflation procedure and you have to add an insole to one of shoes and people have often already swollen feet due to surgery”. (PT 8) |
|  | ***OpenGo Science*** |
|  | - “The insole is easily placed in the shoe and there are no straps or other things around the ankle, that’s nice”. (patient 2) - “The battery connection plate in the insole looks very vulnerable and fragile. And you have to have fine motor skills to put in the coin cell battery”. (patient 2) - “You have to have pockets to carry the smartphone. Thus, that’s a problem because I don’t have pockets right now. And you have to be handy with a smartphone and I myself am not. So that's an issue too, but if someone helps me then it will succeed”. (patient 2) - “It is difficult to preset the exact weight-bearing threshold in the smartphone app with the slider. Apart from using the slider it should be convenient if you could type in the threshold directly”. (patient 3) - “OpenGo Science is easy to use, easy to wear, simple, it works with a Smartphone and nowadays everyone can handle a smartphone. It seems like a good system”. (patient 3) - “It is easy to use, specifically the use of a smartphone is convenient, and everybody has one”. (patient 4) - “I am more or less satisfied with the ease of use, namely, it was quite difficult to set the exact amount of the upper weight-bearing limit in the weight bearing app on the smartphone”. (patient 5) - “I need a person to help me with the smartphone, I’m not used to it”. (patient 6) - “Easy to use system, it is wireless, has no tubes or control unit attached around body parts, just insoles and gives feedback via your smartphone, perfect“. (patient 7) - “It is ideal that you can connect the insoles with your smartphone and nowadays almost everybody has a smartphone. For me it works very nice”. (patient 8) - “Placing the insoles in the shoes goes silky smooth”. (patient 9) - “It is not easy to set the weight-bearing slider on the smartphone application at the precise amount of kilograms”. (patient 9). - “Although there were some pairing problems (connection between the insole and the smartphone), once it worked it was a simple and easy to use system”. (patient 9) - “The insole and smartphone are easy to use in daily practice” (PT 1) - “Although I’m not familiar with the analysis software it is easy to use“. (PT 1) - “OpenGo Science is quickly applied, easy to use and no long explanations are needed for patients. There are no wires attached, just put the insoles in the patient’s shoe and ready to go”. (PT 2) - “Concerning the weight-bearing app, it is a challenge to set the slider for the upper threshold on the exact amount of kilograms. It is working very precisely” (PT 2) - “I’m very satisfied, it is easy and quick to use. Just place the insole in the shoe, turn on the smartphone and there you are”. (PT 3) - “I am very satisfied with the ease of use, there are just a few steps to use the system”. (PT 4) - “I am satisfied with the ease of use of the system. I like such systems, but it has to work properly at all times. It didn’t”. (PT 5) - “Device is easy to use and real-time feedback via the smartphone is good”. (PT 6) - “I’m inclined to watch the amount of kilograms on the smartphone but it is difficult to instruct the patient in PWB and watch the kilograms on the screen simultaneously”. (PT 6) - “Analysis software on the PC seems extremely useful to analyze weight bearing, but I would have liked weigh bearing measurements expressed in kilograms instead of Newton”. (PT 6) - “Device is easy to use during partial weight-bearing instructions and it seems also usable for the rehabilitations at home”. (PT 7) - “I’m very satisfied with the ease of use. I find the system easy to use. The insole fits well in the shoe and there are no wires attached, so that's easy”. (PT 8) - “I am very satisfied with the ease of use. This system is very easy to use. It speaks for itself. It has a good user interface. Furthermore, it is easy to assemble and the insoles can be put easily in the shoes. Thereby it is not uncomfortable for patients, it looks normal, and it uses a smartphone”. (PT 9) - “The weight-bearing application on the smartphone works, but it is difficult to set the WB thresholds slider precisely on the preferred amount. Perhaps it is more convenient if you could simply type in numbers”. (PT 9) |
| **Effectiveness** | ***SmartStep*** |
|  | - “I’m satisfied with SmartsStep, it does what it has to do.” (patient 2) - “It is difficult to feel how much weight you put on your leg by yourself. I think SmartStep is very useful in dealing with this problem”. (patient 3) - “Overall, I’m satisfied, it is useful. But it is not “very satisfied” because there are a lot of steps involved to prepare the system and you cannot see the feedback when you are instructing the patient”. (PT 4) - “Overall, I am more or less satisfied. This device does what it is supposed to do. However, I didn’t like the ease of use”. (PT 5) - “The system helps you to load the leg according to the instructions”. (patient 6) - “I can hear the beeps clearly and it helps you to comply with weight-bearing instructions”. (patient 7) - “I thinks it is a usable system that helps the patient to comply with partial weight-bearing instructions”. (PT 7) - “This system is inconvenient when patients cannot or are not allowed to bend over to their ankles. In this case they need help from somebody else each time they attach or detach the control unit”. (PT 8) - “Overall, I’m more or less satisfied. Although it is a cumbersome system it does what it has to do, provide feedback on weight bearing”. (PT 8) - “I am very satisfied with the degree to which the device helps you with instructing PWB because of the immediate feedback and the upper and lower thresholds”. (PT 9) - “I am more or less satisfied with the monitoring options of the device. I would want to collect data for longer periods instead of the 10 minutes, that is possible with this system”. (PT 9) - “Overall, I am more or less satisfied. In our facility we would like a device that provides feedback on lower and upper weight-bearing thresholds. I am not so satisfied about the ease of use. But I do think you will get used to the device and with some experience it will take less time to prepare the device”. (PT 9) |
|  | ***OpenGo Science*** |
|  | - “Sometimes it is unclear whether you put enough weight on your leg because you are walking with crutches and smartphone is in your pocket”. (patient 1) - “Because of the beeps or vibrations you exactly know when you are loading the leg too much”. (patient 5) - “This device gives me a feeling of safety, I now know when I’m loading to much”. (patient 6) - “It is a pity the smartphone did not store data. I can’t look back in the smartphone how much load I placed on my leg. When I walked with crutches and tried to comply with the weight-bearing instructions I could not manage to also look on the smartphone for the kilograms, I only heard the beeps” (patient 7) - “Although monitoring weight bearing via the PC looks pretty good, I do not understand why you would not combine data collection via the PC and feedback via smartphone. Now it is not possible. When you are collecting data it is not clear for the patient how much he or she loads the affected leg. Combining these functions seems more convenient”. (PT 2) - “The system works very pleasant. It is clear and is quickly applied”. (PT 4) - “Most unfortunately, I think, this system is not always reliable. Occasionally there was a signal loss and no feedback was provided. Potentially, patients could load the leg too much, in absence of feedback, patients could think they are loading properly”. (PT 5) - “When recording patient’s weight bearing the biofeedback from the smartphone is not available, I would have preferred to also use the smartphone’s feedback during the measurements”. (PT 6) - “It is impractical that feedback via the smartphone can not be provided to the patient when the insoles are collecting data or are connected with the PC. Without feedback we immediately see that the patient loads to much”. (PT 7) - “It is a pity that you have to choose between immediate audio/haptic feedback for the rehabilitant by the smartphone and collecting weight-bearing data that can be seen directly or afterwards on the PC”. (PT 9) |
| **Intrusiveness** | ***SmartStep*** |
|  | - “Although you don’t really feel the control unit around the ankle, the idea of having something around the ankle is strange. Especially, when you would go outside. I think I won’t like that”. (patient 3) - “The control unit around my ankle doesn’t bother me.” (patient 5) - "You look like a crook with an electronic ankle bracelet. However, it is easy to use and helps you in your recovery.” (patient 7) - “I think the ankle bracelet around my ankle looks really terrible, it seems a bit like a prison bracelet”. (patient 8) - “On the one hand it is very nice that you get beeps when you're doing well or are not doing well, but on the other hand it is annoying that the device is beeping constantly. Not only for yourself but also for people around you”. (patient 8) - “It is quite a device and it is really visible. When used in practice is not necessarily an issue, but used outside it is”. (PT 3) - “I can imagine that patients would dislike wearing this device around the ankle since it is visible for other people, maybe it sits in the way or is not convenient when you are wearing trousers”. (PT 9) |
|  | ***OpenGo Science*** |
|  | - “You hardly notice you are wearing something and that is important. I think it is important that the materials do not bother you too much. You already have enough discomfort due to your operation or rehabilitation.” (Patient 8) - “You cannot see that you patient is wearing a device, top”. (PT 3) |
